# Knowing Students' Characteristics: Opportunities to Adapt Physical Education Teaching

**DOI:** 10.3389/fpsyg.2021.619944

**Published:** 2021-02-12

**Authors:** Alina Kirch, Melina Schnitzius, Sarah Spengler, Simon Blaschke, Filip Mess

**Affiliations:** Department of Sport and Health Sciences, Technical University of Munich, Munich, Germany

**Keywords:** student characteristics, secondary school, physical education, personality, physical self concept, motivation, interest, motives

## Abstract

Physical Education (PE) aims to convey the joy of exercise and by this educate students to lifelong physical activeness. Student motivation in PE decreases during the school career. This study therefore comprehensively analyzes student characteristics determining motivation in PE: *General Personality Traits, Physical Self-Concept, Achievement Motive, Motives to be physically active*, and *Sports Interest*. This contribution aims to describe students' prerequisites in the PE context by using an aggregated assessment of the abovementioned general plus sport specific characteristics and to detect gender, class, and school type differences. In total, 1,740 German secondary school students (58.1% female, M = 14.39 years) participate in a cross-sectional questionnaire survey. Descriptive analyses and between subjects MANOVAs followed by univariate ANOVAs with pairwise multiple comparison tests are applied. Gender explains the largest proportion of variance across all characteristics. Regarding individual dimensions, genders differed on 12, grades on two and school types on 11 out of 19 dimensions. PE teachers must adapt teaching to different gender dispositions. In general, group differences ascribe special meaning to student perception and teaching behavior. Findings are discussed in terms of their contribution to the research area and their implementation in teaching practice as well as in PE teacher education or professional training, e.g., aligned teaching methods, arranged learning atmospheres, or adjusted content design of PE lessons.

## Introduction

Physical Education (PE) aims to educate students to lifelong engagement in physical activities and to live a healthy lifestyle. Compulsory school PE reaches all school-aged children and promotes physical activity by offering possibilities to exploit the *movement, games and sports* culture, and at the same time personally develop into a competent, literate, and enthusiastic sports person through experiencing *movement, games, and sports* (Siedentop, 2002, 2009; Kurz, 2008; Farias and Hastie, 2016). PE's aim in general and PE's lesson content in particular therefore entail lifelong personal as well as societal relevance.

PE teachers strive to develop and maintain students' enthusiasm for the subject PE but also for physical activity in general, ideally resulting in a state of intrinsic motivation (Rheinberg and Vollmeyer, 2019). This is important as research has shown that physical activity in general (Dumith et al., 2011; Dishman et al., 2018) and motivation for sports (Knisel et al., 2009) decrease from childhood to adolescence—being especially low in teenage years. Reasons provided relate to sexual maturity (Dumith et al., 2011) or to a change and shift of interests away from physical activity in the course of adolescence (Marques and Gaspar de Matos, 2014). Consequently, only 26% of German adolescents (Finger et al., 2018) fulfill the World Health Organization's (2018) recommendations of 60 min daily moderate- to vigorous-intensity physical activity. Further, the World Health Organization (2020) reported an increasing amount of overweight and obese children. Considering these facts, PE's role of transferring knowledge about and enthusiasm for an active and healthy lifestyle becomes more and more important. The Sport Education Model (Siedentop et al., 2020) is a commonly followed approach aiming to provide students with authentic experiences and by this, gain motivation within PE. By taking on roles within learning experiences, students develop personally and internalize the idea of sport.

PE has to highlight different physical activity capabilities and allow students to experience a multifaceted *movement, games, and sports* culture in order to find their individually preferred activity. Students make use of and experience PE's movement offers differently though. PE lessons therefore require an adequate design, which addresses each student appropriately (Powell and Kusuma-Powell, 2011). It is therefore essential to investigate student characteristics in the PE context.

Scientifically examining student characteristics for targeted and sustainable learning processes in school has been prevalent in general educational research (e.g., Drachsler and Kirschner, 2011; Powell and Kusuma-Powell, 2011). Researchers have typically focused on single characteristics (e.g., Personality Traits or Self-Concept) and examined their relationship to, e.g., students' motivation to learn. Also in the PE context, researchers have examined the relationship between single characteristics and student motivation. In order to meet PE's specific peculiarities and requirements with its accompanied inherent experiences, an examination needs to consider not only general but also sport specific characteristics (Beni et al., 2017). Our study therefore addresses the following five characteristics:

(I) General *Personality Traits* as stable individual differences over time and situation, which explain thoughts, behavior, and emotions (Hogan et al., 1996). The five-factor model describes personality in five dimensions (*Conscientiousness, Openness, Extraversion, Agreeableness, Neuroticism*) and has proven its empirical validity in personality research (Rammstedt et al., 2018). Komarraju and Karau (2005) as well as Ljubin-Golub et al. (2019), e.g., have highlighted the relationship between students' non-cognitive personality traits and their motivation to learn. Relationships between students' personality traits and their motivation to learn, and perform in the lesson have also been shown for PE specifically.
(II) *Physical Self-Concept* as sport specific characteristic is an important mediator for physical activity (Jackson-Kersey and Spray, 2013) and motor abilities (Jekauc et al., 2017). Additionally, students' *Physical Self-Concept* is positively related to motivation in PE (Murcia, 2012). The overarching facets of the *Physical Self-Concept* (Braun et al., 2018) can be categorized as *Sports Competence, Physical Self-Esteem*, and *Global Self-Worth*.
(III) *Achievement Motive*, classified into *Hope for Success* and *Fear of Failure*, has intensively been researched in motivational psychology (Rheinberg, 2006) but also offers links for school-based learning (Urhahne, 2008). Students' *Achievement Motive*, e.g., correlates with their learning performance (Tanaka and Yamauchi, 2000) and learning behavior (Schmalt, 2003). With regard to PE, success-oriented students are more willing to exert themselves and reveal greater subject interest than students with a tendency to avoid failure (Streso, 2015).
(IV) *Motives to be physically active* are considered as triggers for physical activity in general (Lehnert et al., 2011). This knowledge influences the design of sport offerings by e.g., tailoring them to the target group (Lehnert et al., 2011), and thus increases the offerings' fit to individual preferences, outside school but also in school PE. Following Gut et al.'s (2019), Kueh et al.'s (2017), or Lehnert et al.'s (2011) understanding, *Motives to be physically active* represent a central benchmark for specifically designing and conducting PE's lesson content. Gut et al. (2019) ascertain the following *Motives to be physically active*: *Contact, Competition/Performance, Distraction/Catharsis, Body/Appearance, Health, Fitness, Aesthetics, and Risk/Challenge*. In German PE, *Motives to be physically active* have found their way into the curriculum as pedagogical perspectives (Neumann and Balz, 2004) and by this, decisively influence teaching behavior.
(V) Interest is also considered decisive for the development of intrinsic motivation in learning situations (Krapp, 2010), as well as in PE in particular aiming to motivate students sustainably. Adolescence is an important period of life's personal interest development (Hofer, 2010; Hoff et al., 2018). Otundo and Garn (2019) highlighted that situational interest as well as need support provided by the PE teacher predicted students' personal interest. If students' learning and performance in PE is driven by their personal *Sports Interest*, learning processes are considered to be more self-determined, voluntarily more frequent, and thorough as well as more sustainable (Gogoll, 2010).

As highlighted, the abovementioned five characteristics—general *Personality Traits* and sport-specific *Physical Self-Concept, Achievement Motive, Motives to be physically active* as well as *Sports Interest*—have already been individually examined. A collective examination is missing but necessary in order to describe students' holistically and derive targeted teaching strategies, which trigger student's motivation in PE. Furthermore, relationships between individual characteristics can be examined.

Further, most of the abovementioned studies only examine small samples restricted to a certain study group, e.g., one age group or school type. A large-scale study covering different grades, school types, and geographical regions is missing in Germany as well as in international research. Such a study will provide (a) a detailed picture by describing students profoundly, and (b) a basis for classing the results with existing research.

Due to different student dispositions, it is essential to compare groups of students, e.g., different genders, grades, and school types. This allows identifying differences, which can become significant in practice and help PE teachers to address students appropriately. Differences in students' characteristics in the PE context between genders, grades, or school types have not been analyzed so far. This knowledge though would affect PE teachers in schools and offer possibilities for PE teacher education at university, e.g., target group-oriented teaching from the outset.

In order to draw reliable and valid conclusions regarding a profound knowledge of students in PE, student characteristics—general *Personality Traits* and sport specific *Physical Self-Concept, Achievement Motive, Motives to be physically active* as well as *Sports Interest*—have to be examined collectively, region-wide across different grades and school types. It is hypothesized that different student groups can be distinctly described by their manifestations in the characteristics. Therefore, this paper aims to comprehensively, in a large Germany-wide sample (a) describe students in the PE context by general and sport specific characteristics triggering motivation, and (b) find out whether characteristics differ with regard to gender, grade, or school type.

## Materials and Methods

### Study Design

The student survey on which this article bases was part of the study *SuM PLuS*. *SuM PLuS* was a Germany-wide study carried out in cooperation with DSLV. It comprised a cross-sectional quantitative questionnaire survey of PE teachers and their students. Participating PE teachers were recruited via DSLV and partners, personal contacts, social media, local press, and educational institutions. After participation, PE teachers could additionally register for the student survey of the study. PE teachers received the student survey material including a standardized instruction. Students took 15 to 20 min to complete the questionnaire in class—online (17.3%) or via paper-pencil (82.7%). Data collection took place from April to December 2018. In total, 40.8% of the questionnaires sent out in paper form were returned. The responsible ministries or school authorities of each participating federal state examined ethical and data protection regulations and approved the study. In addition, schools' administration and a respective legal guardian provided their written consent. Furthermore, the study followed the Declaration of Helsinki. Participation was voluntary and participants could withdraw their consent at any time during the examination.

### Sample

In total, 1,740 secondary school students (58.1% female, *M* = 14.39 ± 1.44 years) from 12 German federal states took part in the study. School types were categorized as follows: (1) lower secondary school (*n* = 830), where students finish with an intermediate school-leaving certificate; (2) higher secondary school (*n* = 753), where students finish with a higher education entrance qualification; (3) comprehensive secondary school (*n* = 500), combining different educational paths, where students finish with either of the two aforementioned qualifications (Maaz et al., 2008).

### Measurements

Students' characteristics were measured via the following five instruments: Personality Traits via BFI-K KJ (Kupper et al., 2019), Physical Self-Concept via (Braun et al., 2018), Achievement Motive via AMS-Sport (Herrmann et al., 2014) derived from Elbe et al. (2005), Motives to be physically active via BMZI-JFEA (Gut et al., 2019), and Sports Interest via Sports Interest (Gogoll, 2010) derived from Kunter et al. (2002). All instruments were validated in samples similar to *SuM PLuS*' sample and obtained satisfactory test quality criteria (Table 1). Besides student characteristics, the questionnaire included sociodemographic data such as gender, grade, and school type.

**Table 1:** Applied scales to measure students' characteristics in physical eduation (PE).

**Construct**	**Inventory**	**References**	**Subscales (items per scale/subscale)**	**Cronbachs α**	**Introductory question** **“Sample Item”** **rating level**
(I) Personality traits	BFI-K KJ (Short version of the big five inventory for children and adolescents)	Kupper et al., 2019	Conscientiousness (6) Openness (6) Extraversion (3) Agreeableness (6) Neuroticism (5)	0.69 0.76 0.90 0.63 0.71	How do you assess yourself and your behavior in everyday life? “I get nervous easily” 5 point scale from 1 = strongly disagree to 5 = strongly agree
(II) Physical Self-Concept	PSDQ-S (Short version of the physical self-description questionnaire)	Braun et al., 2018	Sports competence (3) Physical self-esteem (3) Global physical self-concept (5)	0.87 0.94 0.80	How do you rate yourself and your abilities in general as well as in sports? “Most things I do, I do well” 6 point scale from 1 = strongly disagree to 6 = strongly agree
(III) Achievement Motive	AMS-Sport (achievement motive scale-sport)	Herrmann et al. (2014) derived from Elbe et al. (2005)	Hope for success (5) Fear of failure (4)	0.91 0.87	How do you feel when you are faced with a task in sports? “I enjoy athletic tasks in Physical Education that are slightly difficult for me” 4 point scale from 1 = not right to 4 = totally right
(IV) Motives to be physically active	BMZI-JFEA (the bernese motive and goal inventory for adolescence and young adulthood)	Gut et al., 2019	Contact (5) Competition/performance (3) Distraction/catharsis (4) Body/appearance (3) Health (3) Fitness (3) Aesthetics (2) Risk/challenge (3)	0.87 0.68 0.84 0.85 0.77 0.81 0.67 0.71	Why do you engage in sports in your free time or why would you engage in sports? “To do something in a group” 5 point scale from 1 = strongly disagree to 5 = strongly agree
(V) Sports interest	Sports interest	Gogoll (2010) derived from Kunter et al. (2002)	Sports Interest (3)	0.81	What do you think about sports? “Sport is important to me” 4 point scale from 1 = strongly disagree to 4 = strongly agree

### Statistical Analyses

In the data screening process, accuracy, missing values, and outliers were checked. In descriptive analyses, missing values were excluded case wise. In inferential analyses, 337 participants were excluded list wise due to missing values (Graham, 2009). A total of 1,376 participants remained in the final sample meeting the assumptions for linearity, equality of covariance matrices and absence of multicolinearity (Pituch and Stevens, 2016). Between subjects multivariate analysis of variances (MANOVA) was conducted with independent variables gender (female, male), grade (7, 8, 9, 10), and school type (lower secondary school, higher secondary school, comprehensive secondary school) predicting dependent variables *Personality Traits* (I), *Physical Self Concept* (II), *Achievement Motive* (III), and *Motives to be physically active* (IV). One-dimensional *Sports Interest* (V) was considered in univariate analyses of variance (ANOVA). If MANOVA showed significant results, univariate ANOVAs and, in case of significance, follow-up post hoc tests (Huberty and Morris, 1989) were conducted.

Univariate ANOVAs were used to examine individual dependent variable contributions of the scales' dimensions: (I) *Conscientiousness, Openness, Extraversion, Agreeableness, Neuroticism*, (II) *Sports Competence, Physical Self-Esteem, Global Physical Self-Concept*, (III) *Hope for Success, Fear of Failure*, (IV) *Contact, Competition/Performance, Distraction/Catharsis, Body/Appearance, Health, Fitness, Aesthetics, Risk/Challenge*, and (V) *Sports Interest*. Due to unbalanced data, sums of squares were calculated adaptively following Fox's (2016) recommendations for ANOVA modeling. Last, *p*-adjusted Dunnett–Tukey–Kramer (DTK) (Li, 2012). Pairwise Multiple Comparison Tests were applied in order to show differences between independent variables. RStudio was used (Version 1.2.5033, RStudio Inc., Boston, USA) for data analysis.

## Results

### Overview of Student Characteristics

The following section describes students by their manifestations in the five chosen characteristics. Table 2 shows the sample's score (*M, SD*) in total and differentiated by gender, grade as well as school type.

**Table 2:** Overview of student characteristics—total, gender, grade, and school type.

**Variable**	**Total**	**Gender**		**Grade**				**School Type**		
		**Female**	**Male**	**7**	**8**	**9**	**10**	**Lower**	**Higher**	**Comprehensive**
	***N* = 1,740**	***n* = 1,011**	***n* = 701**	***n* = 424**	***n* = 430**	***n* = 486**	***n* = 400**	***n* = 747**	***n* = 581**	***n* = 375**
	**M ± SD**	**M ± SD**	**M ± SD**	**M ± SD**	**M ± SD**	**M ± SD**	**M ± SD**	**M ± SD**	**M ± SD**	**M ± SD**
**(I) Personality Traits**										
Conscientiousness	3.52 ± 0.77	3.57 ± 0.75	3.45 ± 0.81	3.54 ± 0.79	3.47 ± 0.82	3.53 ± 0.76	3.53 ± 0.73	3.48 ± 0.79	3.58 ± 0.74	3.50 ± 0.78
Openness	3.46 ± 0.63	3.49 ± 0.62	3.41 ± 0.65	3.36 ± 0.65	3.42 ± 0.66	3.49 ± 0.63	3.57 ± 0.57	3.38 ± 0.66	3.57 ± 0.59	3.44 ± 0.63
Extraversion	3.40 ± 1.33	3.31 ± 1.36	3.53 ± 1.29	3.37 ± 1.36	3.50 ± 1.30	3.41 ± 1.33	3.29 ± 1.35	3.36 ± 1.37	3.52 ± 1.27	3.30 ± 1.36
Agreeableness	3.73 ± 0.67	3.72 ± 0.70	3.73 ± 0.64	3.75 ± 0.72	3.73 ± 0.68	3.74 ± 0.63	3.69 ± 0.67	3.62 ± 0.72	3.85 ± 0.61	3.75 ± 0.63
Neuroticism	2.71 ± 0.82	2.89 ± 0.81	2.46 ± 0.77	2.66 ± 0.82	2.71 ± 0.80	2.70 ± 0.82	2.80 ± 0.84	2.80 ± 0.83	2.58 ± 0.80	2.75 ± 0.81
**(II) Physical Self-Concept**										
Sports competence	4.50 ± 1.08	4.32 ± 1.06	4.75 ± 1.06	4.46 ± 1.15	4.55 ± 1.07	4.53 ± 1.04	4.43 ± 1.10	4.35 ± 1.10	4.66 ± 1.02	4.54 ± 1.11
Physical self-esteem	4.28 ± 1.38	4.06 ± 1.45	4.61 ± 1.20	4.38 ± 1.41	4.24 ± 1.44	4.32 ± 1.31	4.20 ± 1.36	4.09 ± 1.44	4.53 ± 1.27	4.29 ± 1.35
Global self-worth	4.59 ± 0.89	4.50 ± 0.92	4.74 ± 0.82	4.51 ± 0.96	4.53 ± 0.92	4.65 ± 0.83	4.66 ± 0.84	4.45 ± 0.90	4.75 ± 0.86	4.65 ± 0.85
**(III) Achievement Motive**										
Hope for success	2.75 ± 0.80	2.63 ± 0.81	2.91 ± 0.75	2.77 ± 0.83	2.74 ± 0.79	2.74 ± 0.78	2.73 ± 0.79	2.63 ± 0.84	2.87 ± 0.74	2.79 ± 0.75
Fear of failure	1.89 ± 0.78	1.99 ± 0.80	1.75 ± 0.73	1.87 ± 0.81	1.94 ± 0.79	1.86 ± 0.75	1.91 ± 0.78	1.95 ± 0.81	1.81 ± 0.76	1.90 ± 0.75
**(IV) Motives to be physically active**										
Contact	2.87 ± 1.24	2.71 ± 1.24	3.10 ± 1.21	3.10 ± 1.32	2.83 ± 1.17	2.81 ± 1.27	2.79 ± 1.19	2.61 ± 1.26	3.14 ± 1.15	2.97 ± 1.25
Competition/ performance	2.83 ± 1.14	2.54 ± 1.04	3.25 ± 1.15	2.80 ± 1.16	2.83 ± 1.08	2.79 ± 1.15	2.91 ± 1.18	2.67 ± 1.16	3.03 ± 1.10	2.85 ± 1.12
Distraction/ catharsis	2.99 ± 1.23	3.02 ± 1.25	2.95 ± 1.21	2.72 ± 1.23	2.84 ± 1.20	3.09 ± 1.27	3.27 ± 1.13	2.84 ± 1.27	3.10 ± 1.16	3.11 ± 1.23
Body/ appearance	2.99 ± 1.36	3.16 ± 1.38	2.75 ± 1.28	2.92 ± 1.39	2.95 ± 1.40	3.00 ± 1.34	3.10 ± 1.29	3.17 ± 1.39	2.80 ± 1.29	2.94 ± 1.34
Health	3.12 ± 1.18	3.10 ± 1.19	3.14 ± 1.18	3.00 ± 1.26	3.06 ± 1.19	3.13 ± 1.20	3.27 ± 1.06	3.14 ± 1.23	3.04 ± 1.14	3.19 ± 1.16
Fitness	3.96 ± 1.01	3.94 ± 1.02	4.00 ± 1.00	3.80 ± 1.12	4.02 ± 0.97	3.97 ± 1.05	4.05 ± 0.87	3.89 ± 1.08	3.98 ± 0.95	4.07 ± 0.96
Aesthetics	2.84 ± 1.23	2.80 ± 1.23	2.89 ± 1.24	2.95 ± 1.27	2.84 ± 1.24	2.77 ± 1.26	2.82 ± 1.16	2.73 ± 1.26	2.98 ± 1.18	2.84 ± 1.24
Risk/ challenge	2.73 ± 1.13	2.53 ± 1.09	3.01 ± 1.12	2.79 ± 1.20	2.68 ± 1.06	2.76 ± 1.18	2.68 ± 1.05	2.66 ± 1.20	2.74 ± 1.04	2.84 ± 1.09
**(V) Sports Interest**										
Sports interest	3.10 ± 0.76	3.01 ± 0.77	3.23 ± 0.72	3.04 ± 0.79	3.11 ± 0.72	3.11 ± 0.74	3.12 ± 0.78	2.93 ± 0.78	3.27 ± 0.69	3.16 ± 0.73

*Number of participants [in total (N); in different groups (n)]; means (M) and standard deviations (SD)*.

### Gender, Grade, and School Type Differences

This section reports differences between students' gender, grade, and school type. Table 3 shows significant differences (*p* < 0.05) in the respective variables, with effect sizes (η^2^) and *post hoc* results between different groups (CI). MANOVA analyses revealed small to large effects (Cohen, 1988) whereas ANOVAs only showed significant differences with small effects.

**Table 3:** Significant differences in student characteristics between students' gender, grade, and school type.

**Variable**	**Gender**				**Grade**									**School Type**^**a**^					
	**P**	**F**	**η^2^**	**m/f**	**p**	**F**	**η^2^**	**7/8**	**7/9**	**7/10**	**8/9**	**8/10**	**9/10**	**P**	**F**	**η^2^**	**HSS/LSS**	**CSS/LSS**	**CSS/HSS**
				**CI**				**CI**	**CI**	**CI**	**CI**	**CI**	**CI**				**CI**	**CI**	**CI**
**(I) Personality traits**	<0.001	25.05	0.09											0.001	5.23	0.04			
Conscientiousness	0.005	7.90	0.01	0.20/0.03										0.047	3.06				
Openness	0.006	7.47	0.01	0.15/0.01										<0.001	14.90	0.02	0.1/0.28		0.24/0.03
Extraversion	0.005	7.96	0.01	0.08/0.36										0.042	3.19				
Agreeableness														<0.001	14.79	0.02	0.13/0.32	0.02/0.24	
Neuroticism	<0.001	85.74	.06	0.51/0.34										0.003	5.96	0.01	0.33/0.10		0.03/0.30
**(II) Physical self-concept**	<0.001	22.69	0.05		0.002	2.98	0.02							<0.001	4.57	0.02			
Sports competence	<0.001	46.34	0.03	0.31/0.54										0.001	7.24	0.01	0.16/0.47	0.02/0.38	
Physical self-esteem	<0.001	45.95	0.03	0.41/0.69										<0.001	9.27	0.01	0.24/0.63		0.47/0.02
Global self-worth	<0.001	19.46	0.01	0.15/0.33	0.027	3.08	0.01							<0.001	11.43	0.02	0.16/0.42	0.05/0.33	
**(III) Achievement motive**	<0.001	21.66	0.03											0.005	3.72	0.01			
Hope for Success	<0.001	35.72	0.03	0.20/0.37										<0.001	8.17	0.01	0.12/0.35	0.04/0.29	
Fear of Failure	<0.001	27.52	0.02	0.32/0.16															
**(IV) Motives to be physically active**	<0.001	28.17	0.14		<0.001	4.04	0.07							<0.001	5.28	0.06			
Contact														<0.001	7.76	0.01	0.35/0.70	0.15/0.56	
Competition/ performance	0.020	5.44	.00	0.59/0.83															
Distraction/catharsis					0.026	3.11	0.01		0.13/0.62	0.30/0.80	0.02/0.49	0.20/0.66							
Body/appearance																			
Health																			
Fitness																			
Aesthetics																			
Risk/challenge	<0.001	9.84	0.01	0.36/0.60															
**(V) Sports interest**																			
Sports interest	<0.001	17.90	0.01	0.14/0.30										<0.001	24.59	0.03	0.24/0.45	0.11/0.35	

Significant differences (p < 0.05); F, ratios of variances; (η^2^), effect sizes; CI, confidence interval.

a *HSS, Higher Secondary School; LSS, Lower Secondary School; CSS, Comprehensive Secondary School*.

#### Gender Differences

According to Table 3, statistically significant main effects of gender occurred in *Personality Traits* [*F*_(5, 1,348)_ = 25.05, *p* = < 0.001, η^2^ = 0.09], *Physical Self-Concept* [*F*_(3, 1,350)_ = 22.69, *p* = < 0.001, η^2^ = 0.05], *Achievement Motive* [*F*_(2, 1,351)_ = 21.66, *p* = < 0.001, η^2^ = 0.03], and *Motives to be physically* active [*F*_(8, 1,345)_ = 28.17, *p* = < 0.001, η^2^ = 0.14]. The multivariate η^2^ implied that 3–14% of multivariate variance of the dependent variables was associated with gender. Univariate analyses yielded significant differences between boys and girls in 12 dimensions. Girls scored significantly higher on *Conscientiousness, Openness, Neuroticism*, and *Fear of Failure* whereas boys scored higher on *Extraversion, Sports Competence, Physical Self-Esteem, Global Self-Worth, Hope for Success, Competition/Performance, Risk/Challenge*, and *Sports Interest*.

#### Grade Differences

Statistically significant main effects of grade were found on *Physical Self-Concept* [*F*_(9, 4,056)_ = 2.98, *p* = 0.002, η^2^ = 0.02] and *Motives to be physically active* [*F*_(24, 4,041)_ = 4.04, *p* = < 0.001, η^2^ = 0.07]. The multivariate η^2^ implied that two to seven percent of multivariate variance of the dependent variables was associated with grade. Univariate analyses yielded significant differences between grades in *Global Self-Worth* and *Distraction/Catharsis*. Only the DTK-Test for *Distraction/Catharsis* revealed significant group differences and showed that older students (grades 9 and 10) scored higher than younger students (grades 7 and 8).

#### School Type Differences

Statistically significant main effects of school type were found in *Personality Traits* [*F*_(10, 2,698)_ = 5.23, *p* = 0.001, η^2^ = 0.04], *Physical Self-Concept* [*F*_(6, 2,702)_ = 4.57, *p* = < 0.001, η^2^ = 0.02], *Achievement Motive* [*F*_(4, 2,704)_ = 3.72, *p* = 0.005, η^2^ = 0.01], and *Motives to be physically active* [*F*_(16, 2,692)_ = 5.28, *p* = < 0.001, η^2^ = 0.06]. The multivariate η^2^ implied that one to six percent of multivariate variance of the dependent variables was associated with school type. Univariate analyses yielded significant differences between school types on *Openness*, where higher secondary school students scored higher than lower secondary school and comprehensive school students. Further, differences occurred for *Agreeableness, Sports Competence, Global Self-Worth, Hope for Success, Contact*, and *Sports Interest* where higher secondary school and comprehensive school students scored higher than lower secondary school students. Further, higher secondary school students scored higher on *Openness* and *Physical Self-Esteem*, and lower on *Neuroticism* than comprehensive school and lower secondary school students.

#### Interaction Effects

Interaction effects were calculated to check if groups influenced each other. An interaction effect of gender and school type [*F*_(16, 2,692)_ = 2.49, *p* = 0.001, η^2^ = 0.03] as well as of grade and school type [*F*_(48, 8,100)_ = 1.49, *p* = 0.016, η^2^ = 0.05] was found on *Motives to be physically active*. Univariate analyses showed no further interaction of individual *Motives to be physically active*. Therefore, the interaction effect can be ignored and subsequently no further post hoc tests exploring the interaction were undertaken.

## Discussion

The study's first aim was to describe students in the PE context by an aggregated examination of general plus sport specific characteristics triggering motivation in PE. Results are compared with existing research considering individual characteristics, in order to classify and interpret the findings. The study's second aim was to find out whether students characteristics differ with regard to gender, grade, or school type. In order to make use of the abovementioned classification as well as detected group differences, possible implications for PE teaching practice as well as professional training and teacher education are highlighted.

### Descriptive Comparisons

The study's results—values as well as order of individual dimensions—considering *Personality Traits* are comparable with national studies using the same scale (Rammstedt and John, 2005; Kupper et al., 2019). *Agreeableness* and *Conscientiousness* values are higher in our sample in comparison to students in the international context (Culjak and Mlačić, 2014; Iimura and Taku, 2018; Lodewyk, 2018; Lau and Jin, 2019). This is in line with Schmitt et al.'s (2007) study comparing adults' Big Five personality traits across different countries and cultures. Therefore, detected findings in this study could result from educational or cultural differences.

*Physical Self-Concept* values are comparable to previous studies, which have used the same scale in a sample consisting of teenagers or young adults (Braun et al., 2018). Similar to Braun et al.'s (2018) as well as Stiller and Alfermann's (2007) sample, students obtain the highest score on *Global Self-Worth*. *Global Self-Worth's* score in this study is lower than in Stiller and Alfermann's (2007) older sample. Students' *Sports Competence* values are higher in comparison to students in the international context (Marsh et al., 2002; Guérin et al., 2004; Garn et al., 2019). However, fifth grade students from the USA (Garn et al., 2019) show higher scores than our study's sample. Cultural differences in relation to, e.g., one's self-perception might have influenced this result. It has to be taken into account that younger students often over-estimate themselves (Lan, 2005; Kolovelonis et al., 2013). Further, USA's organization of youth sports culture where all physical activities are typically offered in schools, possibly allows more opportunities to experience various sports easier of access than in Germany where after school sports are commonly outsourced to sports clubs, and where children's experiences often depend on the regional offering and parental support.

The values of *Achievement Motive's* dimensions are comparable to national and international studies (Herrmann et al., 2014; Streso, 2015).

The strongest expression of the *Fitness* motive is in line with the validation sample (Gut et al., 2019) and another study from Germany (Diehl et al., 2018) as well as with studies from Greece (Zervou et al., 2017), Lithuania (Sukys et al., 2019), and Malaysia (Molanorouzi et al., 2015)—all investigating older samples. Only Kilpatrick et al.'s (2003) American sample attributed less importance to the *Fitness* motive than to *Contact, Competition/Performance, Aesthetics*, or *Risk/Challenge*. This could be due to USA's different design of PE's curriculum emphasizing other motives, e.g., competitive sports games (Shape America, 2014). Another reason might be the fact that the importance of fitness has greatly increased in recent years (Wiklund et al., 2019) while Kilpatrick et al.'s (2003) study dates back several years. Fitness' increasing societal relevance points not only to the meaning of the *Fitness* but also the *Health* motive, which in our study obtained the second highest score. Triggering students' meaning assignment to the *Health* motive paves the way to an active and therefore healthy lifestyle.

Regarding *Sports Interest*,Herrmann et al.'s (2014) Swiss student sample (12–15 years) reveals similar *Sports Interest* values as this study's sample. Gogoll's (2010) sample of older students (17–19 years) reveals lower scores than this study's sample indicating that with increasing age not only motivation but also *Sports Interest* decreases. Further, international comparisons are difficult due to the differences in the operationalization of *Sports Interest*.

### Investigated Group Differences

The fact that girls score higher on *Neuroticism* than boys coincides with the assumption that girls are less confident and more timid than boys are (Danthony et al., 2019). The tendency of girls' higher *Neuroticism* is in line with earlier studies examining *Personality Traits* (Kupper et al., 2019). Further, girls' lower *Physical Self-Concept* matches previous research (Klomsten et al., 2004; Klein, 2017). Klein (2017) additionally highlighted a relationship between *Personality Traits* and *Physical Self-Concept*. The fact that boys' *Achievement Motive* values lie above girls' is compatible to boys' higher self-evaluated *Physical Self-Concept* and lower *Neuroticism*. This again underlines the fact that boys are more confident and venturesome than girls are (Cárdenas et al., 2012). Gender differences might be traceable to the puberty phase, which is a major life event for adolescents. It is associated with many rapid biological, social, and psychological changes (Patton and Viner, 2007). While girls tend to gain body fat during puberty, boys tend to gain muscle mass favoring their sports activities (Waylen and Wolke, 2004). Accompanied physical self-perception is one key correlate of physical activities, especially for girls (Stuart et al., 2005). This explains why girls' characteristics are less advantageous for participation in PE than boys' characteristics. Due to socialization effects, boys are physically tougher, more autonomous, and emotionally stoic (Amin et al., 2018), which may explain gender differences. Socialization effects may also be the reason for boys' higher *Sports Interest*, as males generally are more active than females (Finger et al., 2017). This further implies that a parent of the same sex has a greater role model function than a parent of a different sex (Brouwer et al., 2018). Boys' higher *Sports Interest* could also be traced back to PE's and extracurricular sports' performance as well as goal orientation which matches boys' pronounced *Risk/Challenge* and *Competition/Performance* motive orientation. This further corresponds to boys' higher *Physical Self-Concept* and more distinct *Hope for Success*.

Main effects of grade on *Physical Self-Concept* cannot be used for practical considerations as univariate and post hoc tests did not reveal significant differences (Chen et al., 2018). Whether *Physical Self-Concept* develops over the school career, cannot be answered in this study, due to the cross-sectional design and sample restriction to grade seven to ten. Additionally, further characteristics influencing student development must be considered.

Higher-grade students' stronger orientation toward *Distraction/Catharsis* can possibly be explained by *Distraction/Catharsis's* stress-compensating alignment. Academic-related stress is a major concern of secondary and tertiary students (Pascoe et al., 2020). Therefore, older students facing ongoing normative stressors may appreciate the stress-compensating function of physical activities and therefore enjoy *Distraction/Catharsis*-oriented lessons.

Differences between lower and higher secondary school students emphasize the fact that teachers in higher secondary schools face different student characteristics than lower secondary school teachers. Whether the reason for the difference lies in school-based, family-related, or societal parameters, e.g., cannot be answered in this study. One possible explanation for the detected differences could be the fact that lower secondary school students are less often active in sports clubs (Albert, 2017), and therefore have fewer opportunities to strengthen their *Physical Self-Concept*, train their *Achievement Motive*. or awaken their *Sports Interest*.

*Motives to be physically active* are among all characteristics the most easily addressable in PE teaching practice. Regarding the investigated independent variables, gender explains the most whereas grade explains the least variance. This suggests that the examined characteristics, especially *Personality Traits*, differ between genders but are quite stable within secondary schooling, representing a shorter life period (Neyer and Asendorpf, 2018).

### Implications

#### Aligned Teaching Methods

PE teachers can make use of the detected differences in student characteristics in order to design and conduct PE lessons, which address students appropriately. The fact that girls are more conscientious than boys could imply that they, e.g., need more time to practice. They are more interested in mastering things with confidence and therefore, e.g., benefit from process-oriented rather than product-oriented performance evaluation. Girls' higher *Openness* implies a higher interest and willingness to engage in new contents and teaching methods. PE teachers could thus find it easier to teach girls when trying to follow a broad and multi-perspective curriculum. Further, PE teachers should pay head to this result when offering new contents or new perspectives to boys, e.g., by proceeding in small steps or by granting co-determination and including students' ideas and desires in the lessons. Here, the Sport Education Model represents a valuable approach by bringing students to take up different perspectives via different roles. Considering the abovementioned stable traits therefore facilitates teaching and allows appropriately addressing students. This in turn ideally arises their intrinsic motivation in PE and by this contributes to PE's overarching aim to establish motivation for lifelong physical activities.

Boys' higher *Extraversion* facilitates teaching competition-oriented tasks and contents. Comparatively low scores on *Agreeableness* and *Openness* in lower secondary school students can be considered when, e.g., applying cooperation tasks, creative teaching concepts or offering unknown lesson content.

#### Safe Learning Environments

Lower secondary school students' as well as girls' higher level of *Neuroticism* implies that they particularly require safety in PE lessons. Girls' higher *Neuroticism* plays a crucial role in PE. It has been shown that feeling safe in PE is important for students in general (Albert, 2017). Particularly girls in PE require security against risk, injury, or embarrassment (Brown, 2014; Casey et al., 2014). Considering individual learning progress and process, rather than product-oriented teaching approaches, especially during assessment, can take away fear or uncertainty, and promote security as well as a sense of achievement. Additionally, girls' lower *Physical Self-Concept* should be considered when planning and conducting PE lessons. PE teachers need to create and guarantee a learning setting in which all students feel secure and encounter achievement. Such learning settings allow for valuable experiences, which in turn strengthen students' *Physical Self-Concept* (Schmidt et al., 2013). This can further be promoted by, e.g., considering individual learning progress or applying an optimized feedback culture—e.g., recurring self-, peer-, and teacher evaluation (Conzelmann et al., 2011).

According to PE's educational mandate, students' *Physical Self-Concept* should be maintained or increased in the course of the school career. In order to achieve this aim, PE teachers should be aware that particularly girls and lower secondary school students require *Physical Self-Concept* promotion within safe learning environments.

#### Lesson Design and Tasks

Considering this study's results, tasks with a medium degree of difficulty suit most students best. This consequently triggers their motivation in PE (Engeser and Rheinberg, 2008). Because of a predominant success orientation, PE teachers should make sure that students receive enough time, even when fulfilling easy tasks, before moving on to more difficult tasks.

Another starting point is *Motives to be physically active*, which give direction to the lesson's content and design. The *Fitness* motive appeals the most to students, regardless of gender, grade, or school type. The topic fitness is less centrally presented in Germany's PE curriculum than, e.g., sports games, and therefore plays a subordinate role when planning and designing PE lessons. Addressing this motive in different PE strands, e.g., in gymnastics as well as in athletics or games, empowers students to take part in extracurricular physical activities. Boys are more likely addressed by performance-, competition-, or risk-oriented situations. Girls might not feel adequately addressed in strongly performance- and competition-oriented PE lessons where they have to assert themselves – which is common in PE though (Erdmann, 2008; Lund and Kirk, 2020). Therefore, PE teachers should focus on the values and the order of different *Motives to be physically active* in order to address both genders and pupils who do not correspond to the predominant motivational orientation. It is e.g., as important for girls as for boys to cope with risk experiences and to feel pleasure in doing so. Boys are perhaps more willing to take risks and exceed their individual level of requirements whereas girls may need a more gradual approach. *Distraction/Catharsis's* stress-compensative function can be used in higher and mixed-gender grades in order to find meaning in sport. As the *Health* motive is stronger pronounced in higher grades, aligning PE lessons toward *Health* might support students' lifelong engagement in physical activities.

*Sports Interest* also offers potential for PE teaching, especially because of their close link to intrinsic motivation. Considering this study's results, PE teachers should particularly promote *Sports Interest* among girls and lower secondary school students in order to establish the basis for lifelong engagement in physical activities already in adolescence. In line with girls' desire for safe learning environments, PE teachers' need support becomes especially important also to trigger *Sports Interest* among girls.

The fact that numerous gender differences occurred would initially speak for mono-educational PE, as it might be easier to address students adequately (Hannon and Williams, 2008). Only two *Motives to be physically active—Competition/Performance* and *Risk/Challenge—*but all other characteristics except for *Agreeableness* differed between boys and girls. This indicates that in co-educational PE, teaching behavior or teacher-student interactions might be more important than the lessons' content, which is influenced by the choice of pedagogical perspectives, and therefore by its motive orientation. Considering students' personality development within PE's dual function of education to and through sports, co-educational PE offers developmental potentialities (Hill et al., 2012), e.g., raising students' awareness of thoughtfulness and gender equality. Both, a mono-educational and co-educational organization of PE lessons, offers chances but also problems, which have to be taken into account.

## Conclusion

The presented findings contribute to research as well as PE practice. Students' individual characteristics offer different approaches to influence motivation in PE. The aggregated examination leads to a comprehensive picture of students' in the PE context offering various anchors for targeted teaching.

The study highlights the dimensions' varying manifestation within the examined characteristics. Regarding general characteristics, students show low *Neuroticism* and high *Agreeableness*. Considering sport-specific characteristics, students are rather success-oriented and most attracted by the *Fitness* motive. Further, students obtain high values on *Physical Self-Concept* dimensions as well as on *Sports Interest*. Student groups differ, which allows describing them by the manifestation of the examined characteristics. Gender explains the largest proportion of variance across all characteristics with 12 differing dimensions. School types differ in 11 whereas grades only differ in two dimensions. This indicates the characteristics' relative stability. Predominant differences in *General Personality Traits, Physical Self-Concept, Achievement Motive*, and *Sports Interest* ascribe special meaning to student perception and teaching behavior in comparison to lesson content, which is reflected by fewer differences in *Motives to be physically active*.

Results can raise PE teachers' awareness of the fact that certain groups of students may experience PE differently and require appropriate addressing. Findings are transferred into recommendations for PE teachers in schools and can further affect PE teachers participating in professional courses or prospective PE teachers in teacher education.

## Strengths, Limitations, and Outlook

The study's strengths are its nationwide character and its sample size. This was achieved by the support of DSLV and ministerial approvals in the different federal states. Considering several grades and school types makes the study even more meaningful. The comprehensive understanding of student characteristics provides a wide range of discussable results and implications.

PE teachers registered for and instructed the student survey. This might have influenced students' response behavior trying to please the teacher. Further, no information regarding the exact setting and conditions under which the examination took place can be provided. Students' voluntary participation might have biased the sample. Socio-economic stratification was not considered in order to receive a sample resembling the population. An exact response rate cannot be provided as participants were partly recruited via public advertisement and online participation was possible.

Differences regarding grades and school types are mostly comparable to previous results from studies in Germany investigating characteristics individually. As the examined sample differs from students in other countries, a survey in different countries applying the same survey instrument as in PISA (e.g., OECD, 2019) or HBSC (e.g., Inchley et al., 2016) seems interesting. In addition to analyzing and comparing students' characteristics, one could observe PE teaching and see if applied strategies differ considering cultural specific manifestations of characteristics. This knowledge can contribute to teaching recommendations and possibly have an effect on teaching outcomes, e.g., student motivation or achievement.

In order to decide whether student characteristics develop over the school career, a longitudinal survey—also including primary schools in order to cover students' school career comprehensively, is essential.

As the presented results showed potential patterns and previous studies highlighted relationships between at least some of the investigated characteristics, future work should aim to holistically conceive and describe these relationships by means of students' socio-demographic characteristics. Further, the replication crisis in personality research in combination with occurring small effect sizes, emphasize the need for future studies adopting an accordingly comprehensive approach. Clustering students with similar patterns across the individual characteristics, would reduce the complexity, and by this facilitate additional implications without expecting too much of the individual PE teacher. Easily identifiable and distinguishable student types can help PE teachers to plan and conduct targeted PE lessons, which successfully accomplish PE's educational mandate.

## Data Availability Statement

The datasets presented in this article are not readily available because of educational ministries' permits underlying privacy policies. Requests to access the datasets should be directed to filip.mess@tum.de.

## Ethics Statement

Ethical review and approval was not required for the study on human participants in accordance with the local legislation and institutional requirements. Written informed consent to participate in this study was provided by the participants' legal guardian/next of kin.

## Author Contributions

AK, MS, SS, and FM contributed to conception and design of the study. SB, AK, and MS performed the statistical analysis. FM and SS managed and coordinated the responsibility for the research activity planning and execution. AK wrote the first draft of the manuscript. All authors contributed to the article and approved the submitted version.

## Conflict of Interest

The authors declare that the research was conducted in the absence of any commercial or financial relationships that could be construed as a potential conflict of interest.

## Abbreviations

SuM PLuS: Sportunterricht und Motivation: Personbezogene Faktoren von LehrerInnen und SchülerInnen als Determinanten der Schülermotivation/Physical Education and Motivation: Teachers' and Students' Person-Related Factors as Determinants of Student Motivation
DSLV: Deutscher Sportlehrerverband/German PE teacher association.

## References

[B1] Albert K. (2017). Sportengagement Sozial Benachteiligter Jugendlicher. Wiesbaden: Springer.

[B2] Amin A. Kågesten A. Adebayo E. Chandra-Mouli V. (2018). Addressing gender socialization and masculinity norms among adolescent boys: policy and programmatic implications. J. Adolesc. Health 62, S3–S5. 10.1016/j.jadohealth.2017.06.022 29455715 PMC5817048

[B3] Beni S. Fletcher T. Ni Chróinín D. (2017). Meaningful experiences in physical education and youth sport: a review of the literature. Quest 69, 291–312. 10.1080/00336297.2016.1224192

[B4] Braun A. Martin T. Alfermann D. Michel S. (2018). Überprüfung der Reliabilität und Validität der Kurzform des Physical Self-Description Questionnaire (PSDQ-S) in den Altersgruppen des frühen und späten Erwachsenenalters. Zeitschr. Sportpsychol. 25, 115–127. 10.1026/1612-5010/a000236

[B5] Brouwer S. I. Küpers L. K. Kors L. Sijtsma A. Sauer P. J. J. Renders C. M.. (2018). Parental physical activity is associated with objectively measured physical activity in young children in a sex-specific manner: the GECKO Drenthe cohort. BMC Public Health 18:1033. 10.1186/s12889-018-5883-x 30126399 PMC6102934

[B6] Brown D. (2014). Negative Experiences in Physical Education Class and Avoidance of Exercise. Hays, KS: Master of Science, Fort Hays State University.

[B7] Cárdenas J.-C. Dreber A. von Essen E. Ranehill E. (2012). Gender differences in competitiveness and risk taking: comparing children in Colombia and Sweden. J. Econ. Behav. Org. 83, 11–23. 10.1016/j.jebo.2011.06.008

[B8] Casey A. Hill J. Goodyear V. (2014). PE doesn't stand for physical education. It stands for public embarrassment: voicing experiences and proffering solutions to girls' disengagement in physical education, in Sociocultural Issues in Physical Education: Case Studies for Teachers, eds S. B. Flory, A. Tischler, and S. Sanders (Lanham: Rowman & Littlefield), 37–53.

[B9] Chen T. Xu M. Tu J. Wang H. Niu X. (2018). Relationship between Omnibus and *post-hoc* Tests: an investigation of performance of the F test in ANOVA. Shanghai Arch. Psychiatry 30, 60–64. 10.11919/j.issn.1002-0829.218014 29719361 PMC5925602

[B10] Cohen J. (1988). Statistical Power Analysis for the Behavioral Sciences. Hillsdale, NJ: Lawrence Erlbaum Associates.

[B11] Conzelmann A. Schmidt M. Valkanover S. (2011). Persönlichkeitsentwicklung durch Schulsport. Theorie, Empirie und Praxisbausteine der Berner Interventionsstudie Schulsport (BISS). Bern: Huber.

[B12] Culjak Z. Mlačić B. (2014). The big-five model of personality and the success of high school students in physical education. Croat. J. Educ. 16, 471–490.

[B13] Danthony S. Mascret N. Cury F. (2019). Test anxiety in physical education: the predictive role of gender, age, and implicit theories of athletic ability. Eur. Phys. Educ. Rev. 26, 128–143. 10.1177/1356336X19839408

[B14] Diehl K. Fuchs A. Rathmann K. Hilger-Kolb J. (2018). Students' motivation for sport activity and participation in university sports: a mixed-methods study. BioMed Res. Int. 2018:9524861. 10.1155/2018/9524861 30009179 PMC6020653

[B15] Dishman R. K. McIver K. L. Dowda M. Pate R. R. (2018). Declining physical activity and motivation from middle school to high school. Med. Sci. Sports Exerc. 50, 1206–1215. 10.1249/MSS.0000000000001542 29298219 PMC5953776

[B16] Drachsler H. Kirschner P. (2011). Learner characteristics, in Encyclopedia of the Sciences of Learning, ed N. M. Seel (Boston, MA: Springer), 1743–1745.

[B17] Dumith S. Gigante D. Domingues M. Kohl H. (2011). Physical activity change during adolescence: a systematic review and a pooled analysis. Int. J. Epidemiol. 40, 685–698. 10.1093/ije/dyq272 21245072

[B18] Elbe A.-M. Wenhold F. Müller D. (2005). Zur reliabilität und validität der achievement motives scale-sport. Zeitschrift Sportpsychol. 12, 57–68. 10.1026/1612-5010.12.2.57

[B19] Engeser S. Rheinberg F. (2008). Flow, performance and moderators of challenge-skill balance. Motiv. Emot. 32, 158–172. 10.1007/s11031-008-9102-4

[B20] Erdmann R. (2008). Leisten, leistung, sportunterricht. Sportpädagogik 3, 55–61.

[B21] Farias C. Hastie P. (2016). The Sport Education Model: research update and future avenues for practice and investigation. Rev. Portuguesa Ciências Desporto 2016, 73–96. 10.5628/rpcd.16.01

[B22] Finger J. D. Mensink G. Lange C. Manz K. (2017). Gesundheitsfördernde körperliche Aktivität in der Freizeit bei Erwachsenen in Deutschland. J. Health Monit. 2, 37–44. 10.17886/RKI-GBE-2017-027

[B23] Finger J. D. Varnaccia G. Borrmann A. Lange C. Mensink G. (2018). Körperliche Aktivität von Kindern und Jugendlichen in Deutschland – Querschnittergebnisse aus KiGGS Welle 2 und Trends. J. Health Monit. 3, 24–31. 10.17886/RKI-GBE-2018-006.2

[B24] Fox J. (2016). Applied Regression Analysis and Generalized Linear Models. Los Angeles, CA: Sage.

[B25] Garn A. C. Morin A. J. S. White R. L. Owen K. B. Donley W. Lonsdale C. (2019). Moderate-to-vigorous physical activity as a predictor of changes in physical self-concept in adolescents. Health Psychol. 39:, 190–198. 10.1037/hea0000815 31750675

[B26] Gogoll A. (2010). Verständnisvolles Lernen im Schulfach Sport. Sportwissenschaft 40, 31–38. 10.1007/s12662-010-0104-5

[B27] Graham J. W. (2009). Missing data analysis: making it work in the real world. Annu. Rev. Psychol. 60, 549–576. 10.1146/annurev.psych.58.110405.085530 18652544

[B28] Guérin F. Marsh H. W. Famose J.-P. (2004). Generalizability of the PSDQ and its relationship to physical fitness: the European French Connection. J. Sport Exerc. Psychol. 26, 19–38. 10.1123/jsep.26.1.19

[B29] Gut V. Schmid J. Schmid J. Conzelmann A. (2019). The bernese motive and goal inventory for adolescence and young adulthood. Front. Psychol. 9:2785. 10.3389/fpsyg.2018.02785 30740081 PMC6357923

[B30] Hannon J. C. Williams S. M. (2008). Should secondary physical education be coeducational or single-sex? J. Phys. Educ. Recreat. Dance 79, 6–56. 10.1080/07303084.2008.10598126

[B31] Herrmann C. Leyener S. Gerlach E. (2014). IMPEQT-Studie. Dokumentation der Erhebungsinstrumente. Available online at: http://www.dsbg4public.ch/custom/upload/docs/ylx47akr3f5a7m93ggdl1u3xahen2ju1242g.pdf (accessed October 17, 2020).

[B32] Hill G. M. Hannon J. C. Knowles C. (2012). Physical education teachers' and university teacher educators' perceptions regarding coeducational vs. single gender physical education. Phys. Educ. 69, 265–288.

[B33] Hofer M. (2010). Adolescents' development of individual interests: a product of multiple goal regulation? Educ. Psychol. 45, 149–166. 10.1080/00461520.2010.493469

[B34] Hoff K. A. Briley D. A. Wee C. J. M. Rounds J. (2018). Normative changes in interests from adolescence to adulthood: a meta-analysis of longitudinal studies. Psychol. Bull. 144, 426–451. 10.1037/bul0000140 29494193

[B35] Hogan R. Hogan J. Roberts B. W. (1996). Personality measurement and employment decisions: questions and answers. Am. Psychol. 51:469. 10.1037/0003-066X.51.5.469

[B36] Huberty C. J. Morris J. D. (1989). Multivariate analysis versus multiple univariate analyses. Psychol. Bull. 105, 302–308. 10.1037/0033-2909.105.2.302

[B37] Iimura S. Taku K. (2018). Gender differences in relationship between resilience and big five personality traits in Japanese adolescents. Psychol. Rep. 121, 920–931. 10.1177/0033294117741654 29298590

[B38] Inchley J. Currie D. Young T. Samdal O. Torsheim T. Augustson L. (eds.). (2016). Growing up unequal: gender and socioeconomic differences in young people's health and well-being: Health Behaviour in School-Aged Children (HBSC) Study: international report from the 2013/2014 survey. Copenhagen: World Health Organization Regional Office for Europe.

[B39] Jackson-Kersey R. Spray C. (2013). Amotivation in physical education: relationships with physical self-concept and teacher ratings of attainment. Eur. Phys. Educ. Rev. 19, 289–301. 10.1177/1356336X13495625

[B40] Jekauc D. Wagner M. O. Herrmann C. Hegazy K. Woll A. (2017). Does physical self-concept mediate the relationship between motor abilities and physical activity in adolescents and young adults? PLoS ONE 12:e0168539. 10.1371/journal.pone.0168539 28045914 PMC5207408

[B41] Kilpatrick M. Hebert E. Bartholomew J. (2003). Motivation for physical activity: differentiating motives for sport and exercise participation. J. Sport Exerc. Psychol. 25, S80–S81. 10.3200/JACH.54.2.87-94 16255320

[B42] Klein M. (2017). Self-Concept in adolescents: relationship between sport participation, motor performance and personality traits. Sports 5:22. 10.3390/sports5020022 29910382 PMC5968982

[B43] Klomsten A. T. Skaalvik E. M. Espnes G. A. (2004). Physical self-concept and sports: do gender differences still exist? Sex Roles 50, 119–127. 10.1023/B:SERS.0000011077.10040.9a

[B44] Knisel E. Opitz S. Wossmann M. Ketelhut K. (2009). Sport motivation and physical activity of students in three European schools. Int. J. Phys. Educ. 46, 40–53.

[B45] Kolovelonis A. Goudas M. Dermitzaki I. Kitsantas A. (2013). Self-regulated learning and performance calibration among elementary physical education students. Eur. J. Psychol. Educ. 28, 685–701. 10.1007/s10212-012-0135-4

[B46] Komarraju M. Karau S. (2005). The relationship between the Big Five personality traits and academic motivation. Person. Ind. Differ. 39, 557–567. 10.1016/j.paid.2005.02.013

[B47] Krapp A. (2010). Interesse, in Handwörterbuch Pädagogische Psychologie, 4th Edn. ed D. Rost. (Weinheim: Beltz), 280–290.

[B48] Kueh Y. C. Kuan G. Morris T. (2017). The physical activity and leisure motivation scale: a confirmatory study of the Malay language version. Int. J. Sport Exerc. Psychol. 17, 1–16. 10.1080/1612197X.2017.1321029

[B49] Kunter M. Schümer G. Artelt C. Baumert J. R. Klieme E. Neubrand M.. (2002). PISA 2000: Dokumentation der Erhebungsinstrumente. Berlin: Bildungsforschung.

[B50] Kupper K. Krampen D. Rammstedt B. Rohrmann S. (2019). Kurzversion des Big Five Inventory für Kinder und Jugendliche (BFI-K KJ). Diagnostica 65, 86–96. 10.1026/0012-1924/a000216

[B51] Kurz D. (2008). Der Auftrag des Schulsports. Sportunterricht 57, 1–8.

[B52] Lan W. (2005). Self-monitoring and its relationship with educational level and task importance. Educ. Psychol. 25, 109–127. 10.1080/0144341042000294921

[B53] Lau C. Jin Q. (2019). Chinese students' group work performance: does team personality composition matter? Educ. Train. 61, 290–309. 10.1108/ET-06-2018-0141

[B54] Lehnert K. Sudeck G. Conzelmann A. (2011). BMZI – Berner Motiv- und Zielinventar im Freizeit- und Gesundheitssport. Diagnostica 57, 146–159. 10.1026/0012-1924/a000043

[B55] Li H. (2012). A multiple comparison procedure for populations with unequal variances. J. Stat. Theory Appl. 11, 165–181. 10.2307/2287161

[B56] Ljubin-Golub T. Petričević E. Rovan D. (2019). The role of personality in motivational regulation and academic procrastination. Educ. Psychol. 39, 550–568. 10.1080/01443410.2018.1537479

[B57] Lodewyk K. R. (2018). Associations between trait personality, anxiety, self-efficacy and intentions to exercise by gender in high school physical education. Educ. Psychol. 38, 487–501. 10.1080/01443410.2017.1375081

[B58] Lund J. L. Kirk M. F. (2020). Performance-Based Assessment for Middle and High School Physical Education. Champaign, IL: Human Kinetics.

[B59] Maaz K. Trautwein U. Ldtke O. Baumert J. (2008). Educational transitions and differential learning environments: how explicit between-school tracking contributes to social inequality in educational outcomes. Child Dev. Persp. 2, 99–106. 10.1111/j.1750-8606.2008.00048.x

[B60] Marques A. Gaspar de Matos M. (2014). Adolescents' physical activity trends over the years: a three-cohort study based on the Health Behaviour in School-aged Children (HBSC) Portuguese survey. BMJ Open 4:e006012. 10.1136/bmjopen-2014-006012 25287105 PMC4187657

[B61] Marsh H. W. Marco I. T. Abcy F. H. (2002). Cross-cultural validity of the physical self-description questionnaire: comparison of factor structures in Australia, Spain, and Turkey. Res. Q. Exerc. Sport 73, 257–270. 10.1080/02701367.2002.10609019 12230332

[B62] Molanorouzi K. Khoo S. Morris T. (2015). Motives for adult participation in physical activity: type of activity, age, and gender. BMC Public Health 15:66. 10.1186/s12889-015-1429-7 25637384 PMC4314738

[B63] Murcia J. (2012). Motivation and physical self-concept in physical education: differences by gender. Open Educ. J. 5, 9–17. 10.2174/1874920801205010009

[B64] Neumann P. Balz E. (2004). Mehrperspektivischer Sportunterricht. Schorndorf: Hofmann.

[B65] Neyer F. J. Asendorpf J. B. (2018). Psychologie der Persönlichkeit. Berlin, Heidelberg: Springer. 10.1007/978-3-662-54942-1

[B66] OECD (2019). PISA 2018 Results (Volume I): What Students Know and Can Do. Paris: PISA, OECD Publishing.

[B67] Otundo J. O. Garn A. C. (2019). Student interest and engagement in middle school physical education: examining the role of needs supportive teaching. Int. J. Educ. Psychol. 8, 137–161. 10.17583/ijep.2019.3356

[B68] Pascoe M. C. Hetrick S. E. Parker A. G. (2020). The impact of stress on students in secondary school and higher education. Int. J. Adolesc. Youth 25, 104–112. 10.1080/02673843.2019.1596823

[B69] Patton G. C. Viner R. (2007). Pubertal transitions in health. Lancet 369, 1130–1139. 10.1016/S0140-6736(07)60366-3 17398312

[B70] Pituch K. A. Stevens J. (2016). Assumptions in MANOVA, in Applied Multivariate Statistics for the Social Sciences: Analyses With SAS and IBM's SPSS, eds K. A. Pituch, and J. Stevens (New York, NY: Routledge/Taylor & Francis Group), 219–261.

[B71] Powell W. Kusuma-Powell O. (2011). How to Teach Now: Five Keys to Personalized Learning in the Global Classroom. Alexandria, VA: ASCD.

[B72] Rammstedt B. Danner D. Soto C. J. John O. P. (2018). Validation of the short and extra-short forms of the Big Five Inventory-2 (BFI-2) and their German adaptations. Euro. J. Psychol. Assess. 36, 11–13. 10.1027/1015-5759/a000481

[B73] Rammstedt B. John O. P. (2005). Kurzversion des big five inventory (BFI-K). Diagnostica 51, 195–206. 10.1026/0012-1924.51.4.19524771934

[B74] Rheinberg F. (2006). Motivation. Stuttgart: Kohlhammer.

[B75] Rheinberg F. Vollmeyer R. (2019). Motivation. Stuttgart: Verlag W. Kohlhammer.

[B76] Schmalt H.-D. (2003). Leistungsmotivation im Unterricht: über den Einsatz des LM-Gitters in der Schule, in Diagnostik von Motivation und Selbstkonzept, eds J. Stiensmeier-Pelster, and F. Rheinberg (Göttingen: Hogrefe), 105–138.

[B77] Schmidt M. Valkanover S. Roebers C. Conzelmann A. (2013). Promoting a functional physical self-concept in physical education: evaluation of a 10-week intervention. Eur. Phys. Educ. Rev. 19, 232–255. 10.1177/1356336X13486057

[B78] Schmitt D. P. Allik J. McCrae R. R. Benet-Martínez V. (2007). The geographic distribution of big five personality traits: patterns and profiles of human self-description across 56 nations. J. Cross Cult. Psychol. 38, 173–212. 10.1177/0022022106297299

[B79] Shape America (2014). National Standards & Grade-Level Outcomes for K-12 Physical Education. Champaign, IL: Human Kinetics.

[B80] Siedentop D. (2002). Sport education: a retrospective. J. Teach. Phys. Educ. 21. 10.1123/jtpe.2

[B81] Siedentop D. (2009). Introduction to Physical Education, Fitness, and Sport. Boston: McGraw-Hill.

[B82] Siedentop D. Hastie P. A. Van der Mars H. (2020). Complete Guide to Sport Education. Champaign, IL: Human Kinetics.

[B83] Stiller J. Alfermann D. (2007). Die deutsche übersetzung des physical self-description questionnaire (PSDQ). Zeitschr. Sportpsychol. 14, 149–161. 10.1026/1612-5010.14.4.149

[B84] Streso J. (2015). Leistungsmotivation und Sportunterricht. Eine empirische Analyse zur Ausprägung des Leistungsmotivs von Jungen und Mädchen im Sportunterricht. Dr. phil (Doctoral thesis). Magdeburg: Otto-von-Guericke-Universität Magdeburg, Germany.

[B85] Stuart J. H. B. Sarah H. W. Toni M. O. D. Mary E. N. (2005). Correlates of participation in physical activity for adolescent girls: a systematic review of recent literature. J. Phys. Act. Health 2, 423–434. 10.1123/jpah.2.4.423

[B86] Sukys S. Cesnaitiene V. J. Emeljanovas A. Mieziene B. Valantine I. Ossowski Z. M. (2019). Reasons and barriers for University Students' leisure-time physical activity: moderating effect of health education. Percept. Motor Skills 126, 1084–1100. 10.1177/0031512519869089 31407961

[B87] Tanaka A. Yamauchi H. (2000). Causal models of achievement motive, goal orientation, intrinsic interest, and academic achievement in classroom. Shinrigaku Kenkyu 71, 317–324. 10.4992/jjpsy.71.317 11140252

[B88] Urhahne D. (2008). Sieben Arten der Lernmotivation. Psychol. Rundschau 59, 150–166. 10.1026/0012-1924.59.3.150

[B89] Waylen A. Wolke D. (2004). Sex 'n' drugs 'n' rock 'n' roll: the meaning and social consequences of pubertal timing. Euro. J. Endocrinol. 151, U151–U159. 10.1530/eje.0.151u151 15554900

[B90] Wiklund E. Jonsson E. Coe A.-B. Wiklund M. (2019). ‘Strong is the new skinny': navigating fitness hype among teenagers in northern Sweden. Sport Educ. Soc. 24, 441–454. 10.1080/13573322.2017.1402758

[B91] World Health Organization (2018). Physical Activity. Available online at: https://www.who.int/en/news-room/fact-sheets/detail/physical-activity (accessed October 20, 2020).

[B92] World Health Organization (2020). Obesity and Overweight. Available online at: https://www.who.int/en/news-room/fact-sheets/detail/obesity-and-overweight (accessed October 20, 2020).

[B93] Zervou F. Stavrou N. A. M. Koehn S. Zounhia K. Psychountaki M. (2017). Motives for exercise participation: The role of individual and psychological characteristics. Cogent Psychol. 4:1345141. 10.1080/23311908.2017.1345141

